# Testing the Level of Cortisol in Dogs

**DOI:** 10.3390/ani15091197

**Published:** 2025-04-23

**Authors:** Kamila Kaszycka, Małgorzata Goleman, Wanda Krupa

**Affiliations:** Department of Ethology and Wildlife Management, University of Life Sciences in Lublin, 20-950 Lublin, Poland; kamila.kaszycka@up.lublin.pl (K.K.);

**Keywords:** dog, cortisol, blood, saliva, hair, faeces, urine, claws, foetal fluids

## Abstract

This paper focuses on cortisol levels in different dog matrices, emphasising the importance of non-invasive sampling for animal welfare and research ethics. The study reviewed over 18,000 articles from various databases, identifying 78 relevant studies that met the inclusion criteria. The findings reveal a growing trend for non-invasive matrices like saliva, hair, and urine, reliably reflecting acute and chronic stress responses while minimising animal stress during sampling. Other sample types, such as blood, are becoming less and less used due to their stress-inducing factors. Such an approach aligns with the growing concerns regarding animals’ welfare during research design. Each sample type contributes a unique perspective, and the matrix choice depends on the research objectives. Rather than one being universally superior or more reliable, they offer complementary data that enhance our understanding of the animal’s stress profile. Cortisol concentrations may be influenced by many factors, from environmental ones, such as living conditions, to genetic and social ones. This analysis focuses on the challenges often met during cortisol concentrations research, such as small sample sizes and lack of official assessment protocols. In the future, researchers should focus on an interdisciplinary approach by incorporating behavioural measures, other physiological indicators, and refining non-invasive methods. Those practices would enhance the understanding of canine welfare evaluation and promote a more ethical approach in clinical and practical settings.

## Simple Summary

This paper focuses on the different methods of cortisol assessment, highlighting the increasing use of non-invasive methods for cortisol assessment. After reviewing over 18,000 studies, 78 relevant ones were identified, clearly showing a shift towards less harmful methods. The ethics committee recommends that research methods remain as non-invasive as possible, stressing the importance of minimalizing discomfort for research animals. Invasive methods may compromise animals’ welfare and influence physiological parameters. The employment of non-invasive methods of sampling could enhance the reliability of the results by avoiding confounding effects related to stress-induced hormonal changes. Many factors, including environmental, genetic, and social ones, influence cortisol levels. Challenges such as small sample sizes and inconsistent protocols are still a major concern. This study calls for interdisciplinary research combining behavioural and physiological measures, which will help refine different cortisol measurement techniques. Future findings may improve major concerns regarding canines’ welfare and ensure ethical research practices.

## 1. Introduction

**Figure 1:**
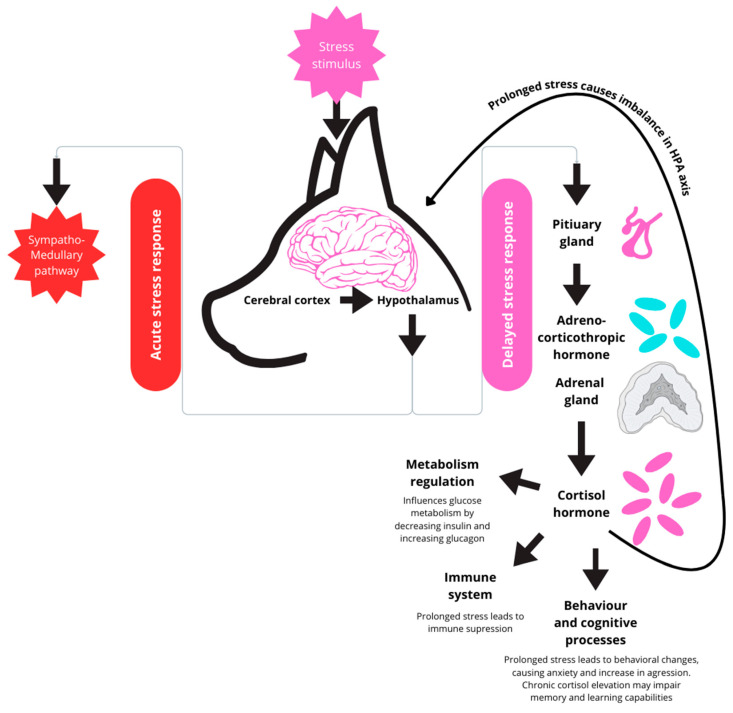
Mechanisms of acute and chronic stress responses in dogs.

Cortisol plays a key role in stress responses in many species, including dogs [1]. While, in the short term, cortisol mobilises the energy levels and various systems to respond to the immediate threat, prolonged cortisol elevation often leads to an impaired immune system and abnormal behaviour, leading to anxiety and other changes, as shown in Figure 1. Researching proper measurement methods and reference ranges helps assess their health, behaviour, and welfare. Dogs have become a significant research concern, as companion animals that share almost all of their environment with humans. Proper care has become a priority for many owners. As dogs increasingly fulfil roles as companions, working, and therapy animals, accurately measuring and interpreting their cortisol levels offer valuable insights into their well-being [2]. Animal welfare is becoming an increasingly popular research topic and a societal problem. Researchers are trying to find new ways to assess it. Society has put a strong emphasis on animal welfare both during research and everyday life. It aligns with minimising stress and harm during research by humanely treating the subjects. This approach is fundamental during the welfare assessment, allowing stress markers to remain unchanged.

Many different cortisol assessment methods have been developed, but each has advantages and limitations. Some of them, like plasma and serum cortisol assessments, although providing immediate cortisol levels, could be a source of great stress and are considered quite invasive. Salivary cortisol measurement is a reliable, non-invasive alternative that reflects the free cortisol levels while minimising sampling-induced stress. Recently, cortisol analysis in alternative matrices, such as hair and claws, has gained attention as a part of chronic stress assessment. Invasive methods can elevate stress levels and impact the physiological response. Therefore, it could compromise the results.

This article explores methods used to assess cortisol levels in dogs, emphasising the importance of non-invasive techniques in promoting ethical research practices and improving animal welfare. Examining the strengths and limitations of these approaches prioritise the health and welfare of the animals they study.

## 2. Materials and Methods

### 2.1. Protocol and Search Strategy

This paper was conducted from November 2023 to December 2024 in the databases Science Direct, PubMed, Web of Science, Taylor & Francis, and Springer and search engine Google Scholar. Said sources were searched using the following keywords:

- Canine” & “Cortisol” “Steroid hormones” & Saliva, and their variations
- “Dog”/“Canine” & “Cortisol”/“Steroid hormones” & Blood/Plasma/Serum, and their variations
- “Dog”/“Canine” & “Cortisol”/“Steroid hormones” & Hair/Coat/Fur, and their variations
- “Dog”/“Canine” & “Cortisol”/“Steroid hormones” & Faeces/Urine, and their variations
- “Dog”/“Canine” & “Cortisol”/“Steroid hormones” & Claw/Nail, and their variations
- “Dog”/“Canine” & “Cortisol”/“Steroid hormones” & Fetal fluid/Amniotic fluid/Allantoic fluid, and their variations
- “Dog”/“Canine” & “Cortisol”/“Steroid hormones” & Cerebrospinal fluid and their variations

That resulted in 60 combinations in every database and search engine. Then, selected by the keywords, article abstracts were read independently by the coauthors and chosen by the eligibility criteria. All articles were also selected by the type of paper—reviews or originals. Only studies in English were included.

### 2.2. Eligibility Criteria in Abstracts

To fit the inclusion criteria, articles had to:

1. be an original paper;
2. be written after 2000 A.D.;
3. include the cortisol matrix material from dogs (or cortisol metabolites).

Exclusion criteria:

1. lack of statistical analysis;
2. less than six cortisol samples taken.

### 2.3. Data Extraction

Data from all the articles have been extracted onto an Excel sheet. The Excel sheet included data like the first author’s name, journal in which the article was published, year of publication, databases in which the article was found, keywords, doi, and passing the criteria in the abstract and full-text screening.

## 3. Results

Over 18,000 articles were searched through, but only 111 passed the first screening based on keywords.

**Figure 2:**
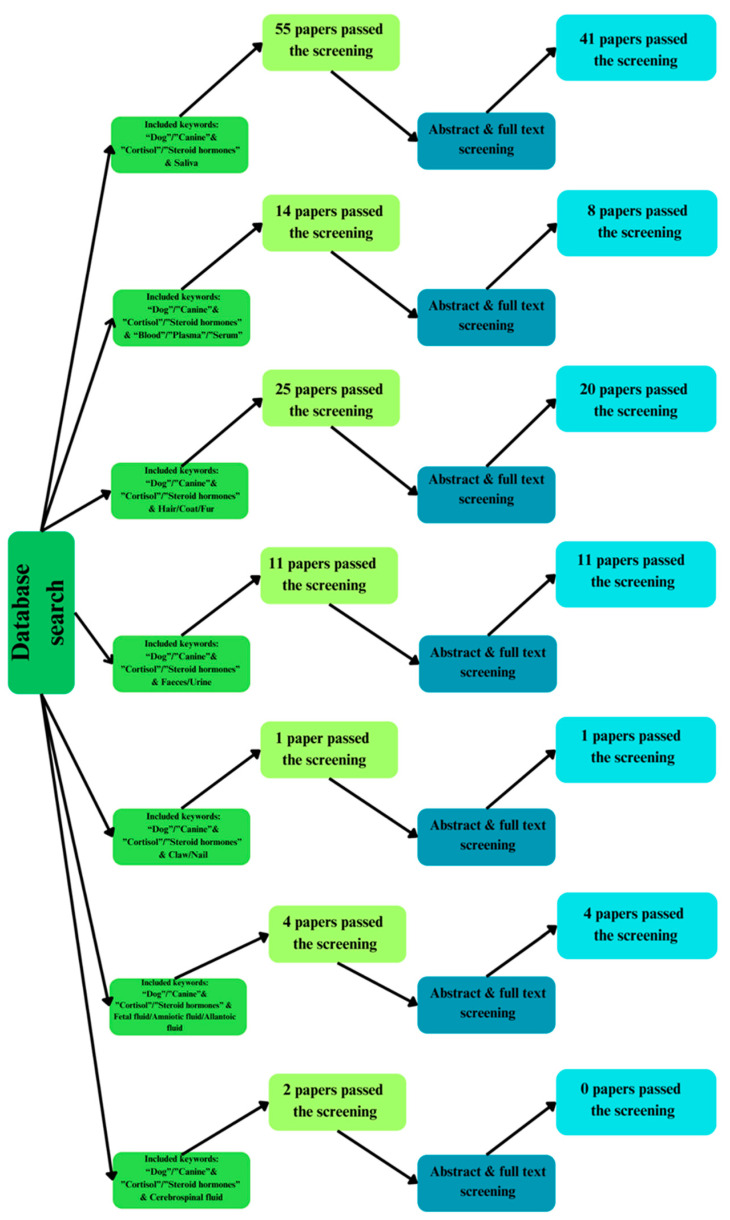
Research phases and articles found according to the keywords and articles found.

Articles found in different stages of the search protocol are shown in Figure 2. They were divided into groups based on the sample material and searched according to keywords. After full screening, 78 independent research papers were found, not including overlapping papers. The abstract and full-text screening pass ratios were widely different between categories. All urine/faeces papers made it through, but none of the cerebrospinal fluids passed the criteria. According to the keyword search, saliva and hair were the most frequently studied sample types, and many passed both screening stages. While blood/plasma/serum sample-type research was from a moderate number of studies, they had a notable drop-off after full-text reviews. Research papers regarding claws, foetal fluids, and cerebrospinal fluids were severely limited.

**Figure 3:**
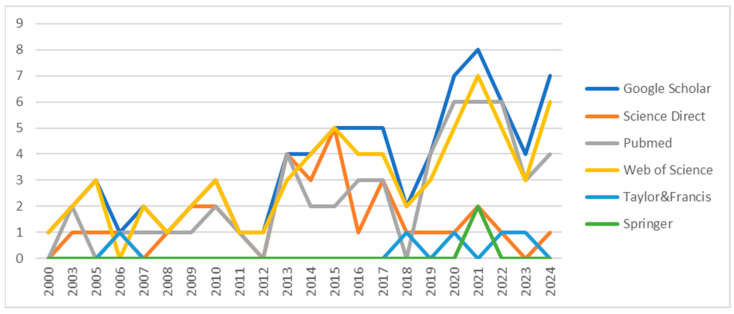
The number of articles in the search bases and research engines per year.

**Figure 4:**
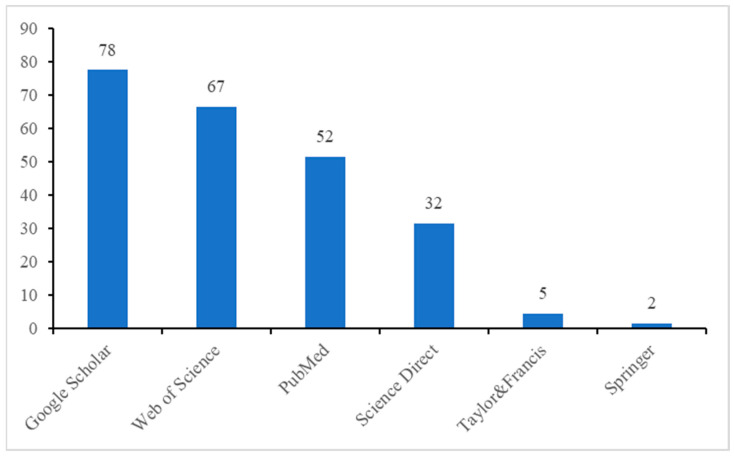
Articles that were able to be identified in each of the bases and research engines.

Figure 3 and Figure 4 show a significant increase in articles fitting the keywords in different bases, especially between 2013 and 2024. Google Scholar and Web of Science showed the highest number of retrieved articles, especially after 2019, and proved to be the most productive sources regarding cortisol research. Meanwhile, Taylor & Francis and Springer had limited representation, suggesting fewer indexed cortisol-related articles in these databases. The disparity in article counts suggests that different databases index different types of research papers, making multi-database searches essential for comprehensive literature reviews.

**Figure 5:**
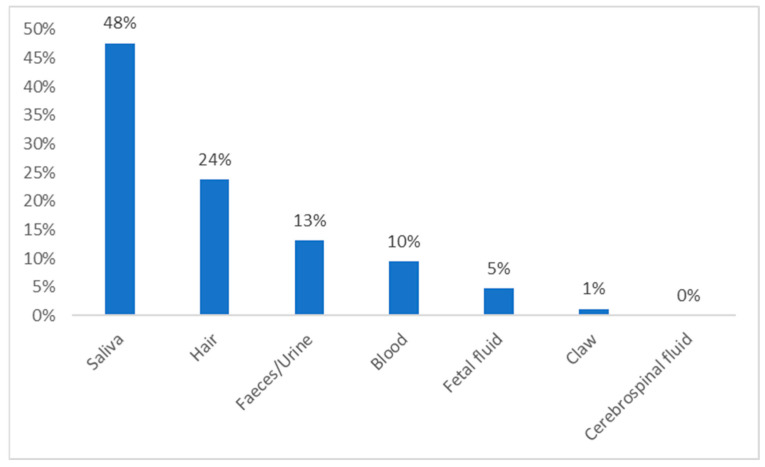
Proportion of articles that passed the final screening, defined by different biological samples.

As Figure 5 shows, less invasive methods, such as saliva, hair, and faeces or urine collection, were published more often than more invasive ones, such as blood or foetal fluids. Claws are one of the newer cortisol matrices in research held on dogs. Therefore, the number of articles does not match that of other methods.

Research with groups of dogs kept as a “normal” or “control” group, privately owned or shelter dogs, not put into extensive stressful situations or physical conditions are shown in Table 1, Table 2, Table 3, Table 4 and Table 5.

## 4. Discussion

There has been a significant shift in cortisol research methods in recent years. More invasive procedures, like blood, cerebrospinal, or foetal fluid matrices, are becoming less popular. For the sake of welfare, and due to major concerns regarding research on animals, the 3Rs (rule—Replacement, Reduction, and Refinement) methods that cause less harm are gaining in popularity. Invasive methods, such as drawing the blood, could be a stressful situation for some of the dogs, especially the ones having anxiety issues during the procedures. Such prolonged situations can impact the stress hormones.

During the data collection, a few significant issues occurred. One of the major issues was the lack of results coming up after typing in the base keywords. Therefore, the search criteria were adjusted several times in the database and the research engine’s check. Some journals prohibit exact keyword matches in titles, which can result in incomplete or inaccurate search results. Another issue is the small number of dogs researched in single papers. Although it aligns with the 3R rule, which is extremely important during invasive procedures, it raises significant concerns regarding the quality of the research. Small sample sizes could lead to insufficient statistical power, meaning there is a higher risk of errors. With fewer subjects, even substantial biological differences in cortisol levels may not be statistically significant, potentially masking fundamental physiological changes. This variability also restricts the generalizability of the findings, as the results might reflect specific conditions of the sample rather than the population of dogs.

### 4.1. Reliability and Invasiveness of Cortisol Measurements

Blood sampling can be invasive and stressful for dogs. Lately, a novel device has been used for collecting blood samples—Fluispotter, a serial venous blood sampling device. This type of blood sampling does not raise cortisol levels in the blood later, and it is not incredibly invasive for dogs. Unfortunately, the implementation of the device demands a short surgical procedure. Other than slight skin irritation in the shaved area, the authors of the study have not noticed any reactions [19].

Cortisol is measurable in blood, saliva, milk, and hair, among other matrices. Cortisol levels differs among them—in dams, it is the highest in plasma and the lowest in milk. Saliva and hair cortisol assessments are reliable methods, but cortisol assessment in milk may demand more sensitive methods than are currently being used [20].

Non-invasive matrices, such as hair and saliva, have emerged as reliable indicators of hypothalamic–pituitary–adrenal (HPA) axis activity, reflecting chronic and acute stress responses. According to Accorsi et al. [21], cortisol in faeces and hair reflects hypothalamic–pituitary–adrenal axis activity and is significantly correlated in those two matrices. Cortisol metabolites (CMs) are the researched factor in faeces. The median CM concentration in shelter dogs was 8.6 ng/g [22]. Hair is one of the most reliable cortisol matrices due to its stability if stored properly. Hair sampling is minimally stressful compared to other popular sampling types [23]. Hair cortisol levels reflect chronic stress rather than just short-term responses. This suggests that measuring cortisol in hair can be a reliable biomarker for assessing a dog’s overall stress levels and temperament [10]. Mincing length can also impact the extracted cortisol levels; the shorter the hair was minced, the higher the cortisol concentration obtained. The outer coat is more prone to washing out and to UV radiation than the undercoat, which can potentially cause lowered cortisol concentrations in the hair [23]. In gathering hair samples, clippers were more successful than scissors. Sampling using clippers demanded less restraint of the animals, and the samples were gathered faster. Despite that, clippers sampling may raise some concerns regarding contamination if not cleaned properly between every animal. Samples collected by razors were poor quality, and the cortisol evaluation was impossible [12].

Salivary cortisol concentrations are correlated to plasma ones, reflecting HPA axis response to acute stressors [24,25]. During the day, salivary and serum cortisol concentrations change, undergoing the same peak trends. Salivary cortisol accurately represents HPA axis activity, presenting approximately 10% of the serum cortisol levels, indicating that salivary cortisol may be helpful as a free cortisol estimation method [26]. Salivary cortisol levels represent a reliable indicator of acute stress across different life stages and stress situations in dogs. After the stress tests, the salivary cortisol concentrations rose significantly in both puppies and adult females [27].

Cortisol variability (iCV) in saliva can be a useful indicator of a dog’s coping mechanism. Higher iCV is associated with better HPA axis regulation and adaptive stress coping, while lower iCV may reflect constrained cortisol responses, potentially indicating chronic stress [28].

When it comes to welfare assessment, neither behavioural observations nor salivary cortisol levels should be the only tool; both should be taken into account [29]. Dog behaviour and body language could be helpful in chronic stress assessment but only if they are combined with physiological markers due to individual differences and easy misinterpretations [1].

Saliva collection has many limitations that are not always considered when assembling the studies. The sampling swab’s material or flavour can impact the cortisol concentrations and the sample size. Beef-flavoured ropes induced unpredictable variability in the cortisol assessment. While low citric acid concentration did not have the same effect, it also did not increase the size of the samples. As a swab material, both hydrocellulose and cotton were equally effective [30]. Filter paper can be one of methods used for gathering saliva samples; it is not costly and avoids the influence of cotton ingredients presented in cotton swabs. Saliva cortisol concentrations correspond to rises in plasma. If the salivary sample was taken for longer than 2 min, the cortisol levels in the samples increased significantly if the dogs were not used to brushing their teeth. In dogs used to brushing, saliva collection by filter papers could be a low-stress method of gathering samples [31].

Flavoured swabs can increase the saliva production, especially if enriched with ginger. Ginger Salimetrics swabs provided almost three times as much saliva as the plain control group, without negatively impacting the pH levels. More prolonged exposure to ginger swap flavour heightened the cortisol concentrations in saliva, but the effect did not occur in 30-s exposure to the flavoured swab. In all saliva samples, cortisol corresponded with serum cortisol [32].

Up to 4 min after a stressful situation for the dogs, the salivary cortisol levels did not increase significantly; therefore, restricting the dog for the sampling procedure should not impact the results at that time. The cortisol values varied between the dogs and over time; therefore, taking multiple samples over several days could yield more reliable results [33].

Storing samples can be problematic. Keeping blood samples in freezers at −20 °C did not significantly impact the cortisol levels, although storing them at 4–5 °C lowered them by about 12.5% [34].

Urine sampling is non-invasive and could be part of canine welfare assessment after the sampling method is standardised [35]. Because a urinary assessment is less invasive than blood tests, it could be more convenient and accurate in diagnosing hypercortisolism than the currently used diagnostics [36]. Unfortunately, not all methods of cortisol evaluations are entirely reliable, especially at low urinary concentrations. Methods and different laboratories can differ when it comes to reliability; therefore, for the diagnosis of different conditions, veterinarians should interpret the results with caution [37].

One of the cortisol metabolites (CMs) in the urine assessment procedure, by using antibodies, is not always reliable. Antibody B, which works against cortisol-21-hemisuccinate, provides more precise and accurate cortisol measurements. Antibody A, raised against cortisol-3-carboxymethyl-oxime, could lead to false high results, which may mislead veterinarians when diagnosing Cushing’s syndrome in dogs. Due to the low specification in the free cortisol, the results can be falsified by cortisol metabolites. Proper cortisol assessment in urine may demand an additional extraction procedure [38].

Cortisol concentrations in healthy puppies are higher in allantoic than amniotic fluids, although the increase is tied to each other [39]. The amniotic and allantoic cortisol concentrations did not differ, even when drugs used during delivery, such as propofol and alfaxalone, were considered [40]. Amniotic fluid parameters might help predict neonatal outcomes in dogs [18].

### 4.2. Health, Genetic, and Behavioral Influences on the Cortisol Levels

#### 4.2.1. Age

Most papers do not signify an age-related difference in cortisol concentrations [4,12,20,41,42,43,44]. Other research, focused on different age groups, has reported different cortisol concentrations, especially in puppies’ saliva. This effect decreased with age [27,45]. Despite no major differences in the samples taken before the Strange Situation Test (SST) between aged and adult dogs, after the strange situation test (SST), senile dogs had increased salivary cortisol. The difference was explained by less successful coping with mild social stress in senile dogs [24]. According to McCullogh et al. [46], older dogs had significantly lower salivary cortisol levels.

#### 4.2.2. Sex

Most papers do not signify a difference in cortisol levels in different matrices between males and females [8,11,16,41,42,43,44,47]. Individual cortisol variations in saliva also did not differ according to sex [28]. In some breeds, Park et al. [48] found a significant effect of sex on cortisol levels in hair. Bowland et al. [2] also found that females have a higher cortisol level. Wojtaś et al. [44] showed that search and rescue male dogs had slightly higher cortisol levels during open field examination. Lower cortisol concentrations in females was also confirmed by McCullough et al. [46]

#### 4.2.3. Breed and Fur Colour

Data regarding hair colour in the assessment of hair cortisol concentrations (HCCs) differ. According to Bowland et al. [2], hair colour can impact cortisol levels—dark and mixed fur had lower cortisol levels than light fur. Therefore, it should be considered when comparing research groups. Maxwell et al. [8] claimed that white-haired dogs had significantly higher cortisol levels than black-haired dogs in the Korean Jindo breed. Other researchers have argued with that, not finding hair colour as a significant variable in hair cortisol concentrations [14].

In puppies, cortisol levels in their claws (shown in Table 4) and hair (65.2 ± 52.23 pg/mg) are correlated and not influenced by breed size [11,12,16]. Breed did not affect the salivary cortisol concentrations [41]. According to other researchers, breed can have a major impact on cortisol concentrations in different matrices. Wojtaś et al. [44] also claimed that breed had an impact on the salivary cortisol levels. The effect can be caused by the body weight of said breed or HPA axis ability to regulate predispositions. Smaller dogs were more stressed than large and giant ones, which could be tied to their greater sensitivity to the environment [49]. Sometimes, even providing saliva samples in small breeds can be difficult [45]. Shin and Shin [4] found no significant differences in cortisol concentrations among the groups based on weight.

#### 4.2.4. Time

During the day, the salivary and serum cortisol concentrations change, undergoing the same peak trends. At sunrise, the cortisol levels in both matrices increase, reaching their peak in the middle of the photo phase [26]. While sheltered dogs’ cortisol levels rise significantly in the mornings, probably due to higher arousal tied to feeding time and human companionship, pet dogs and dogs kept in pens presented the highest salivary cortisol levels in the evenings [49]. Samples of saliva collected in the mornings had higher cortisol concentrations than those collected during any other time of the day [42].

In other papers, no circadian rhythm was found in the salivary cortisol concentrations, and there was no statistically significant increase in cortisol in samples taken at different hours of the day [25,43,50].

Siniscalchi et al. [10] found different levels of cortisol in hair samples taken during different times of the day. Since hair cortisol reflects chronic stress, the effect is unexplainable.

#### 4.2.5. Health, Genetic Predispositions and Diet

Dog’s health and experienced stressors during illness can majorly impact and dysregulate the HPA axis. For example, malnourishment can increase cortisol levels in hair as a significant long-term stress factor [2]. On the contrary, there was no difference in the hair cortisol levels found in healthy and chronically ill ones, despite the higher probability of impaired welfare levels due to the physical and physiological effects of illness [14].

Dogs that have suffered traumatic injury had a significantly higher cortisol/creatinine ratio in their urine within 24 h after a road traffic incident than the ones without traumatic injury history. The increase in cortisol corresponded with the severity of the trauma. A higher cortisol/creatinine ratio was also noticeable in non-survivor dogs [51].

Premature born puppies had significantly higher cortisol levels in both hair and claws compared to term-born dead puppies or those that died within 1–30 days [16]. Amniotic fluid cortisol was higher in puppies that had not survived 24 h after birth [39]. According to Groppetti et al. [18], the amniotic fluid cortisol concentrations were higher in stillborn puppies, although it was not correlated to the Apgar score in infants born alive. Lower cortisol concentrations in amniotic fluids were correlated with higher Apgar scores in healthy puppies and with delivery with ELCS. Those cortisol concentrations may have been affected by the puppy’s and mother’s HPA axis activity. Amniotic cortisol concentrations may be considered a part of the assessment of a newborn’s evaluation in the future [17].

In dogs with Canine Atopic Dermatitis (CAD), the hair cortisol levels were higher than in healthy dogs [13,48]. This could be caused by prolonged stress or physical discomfort caused by illness [48]. The more severe CAD cases the dogs suffered from, the higher their HCC. Heightened cortisol levels might have been tied to the chronic discomfort those dogs experienced [13]. Suffering from CAD can significantly impact dogs’ welfare. The correlation between HCC and pruritus highlights the psychological toll of chronic itchiness in dogs. Treating CAD with lokivetmab improves dogs’ quality of life, which is reflected in HCC after the second treatment. Reducing pruritus alleviates physical discomfort and mitigates chronic stress [52].

Dogs suffering from idiopathic epilepsy had significantly lower cortisol levels than a control group of healthy dogs. Some medications taken for epilepsy can lower cortisol levels [53].

In brachycephalic breeds, brachycephalic obstructive airway syndrome (BOAS) is common due to their specific anatomical predispositions. Said syndrome affects dogs’ physical abilities. Dogs suffering from BOAS have shown no significant response in salivary cortisol levels compared to dogs from the same breeds and control group that had not been affected. Those results might lead to the conclusion that, in dogs with BOAS, the HPA axis is dysregulated and has a decreased responsiveness, since the cortisol levels had not increased after a physically demanding situation [54]. Another study found no statistically significant difference in fur cortisol concentrations between the BOAS-affected group (median: 0.99 pg/mg) and the control group with mild or no BOAS signs. This result indicates that a single fur cortisol measurement cannot reliably distinguish between dogs with and without clinically significant BOAS. Additionally, regardless of their dogs’ BOAS status, none of the owners reported signs of chronic stress in their pets. This implies that owners may not fully recognize or may underestimate the stress related to BOAS. A comprehensive clinical examination by a skilled veterinarian remains the most reliable method for diagnosing BOAS, as fur cortisol measurements alone are not sufficient to assess chronic stress in these dogs [55].

Hypercortisolism, also known as Cushing’s syndrome, is a condition characterized by prolonged exposure to high levels of cortisol. Cortisol metabolites, such as 6β-hydroxycortisol, can be valuable tools in the hypercortisolism diagnostic process when urinary lactate is considered. Those markers could differentiate Cushing’s syndrome from nonadrenal diseases [36]. Dogs suffering from hypercortisolism had significantly higher salivary cortisol levels than healthy dogs, which could serve as a part of a non-invasive diagnostic procedure in the future [25]. In dogs affected by hypercortisolism, trilostane can reduce cortisol levels, improving clinical symptoms. However, since some dogs developed trilostane-induced hypocortisolism, the doses may need to be adjusted over time [56]. The low dosage of dexamethasone is often used as a part of the diagnosis for Cushing’s Syndrome, as it leads to the suppression of endogenous cortisol levels in healthy dogs. In dogs with hyperadrenocorticism, said negative feedback is ineffective mainly due to the independent cortisol secretion. In healthy beagles, dexamethasone suppressed the plasma cortisol levels for longer than previously expected. In all dogs, cortisol was suppressed from 4 to 8 h, but some individuals had lowered levels even after 48 h since drug administration. A 72-h period was deemed safe for the repetition of a low-dose dexamethasone suppression test [57].

Genetics can play a factor in cortisol responses to stressors. P-glycoprotein, encoded by the MDR1, a gene responsible for multi-drug resistance in dogs, is an efflux carrier that generally restricts the transfer of cortisol through the blood–brain barrier. In dogs with MDR1 mutation, the P-gp is absent, leading to increased cortisol access to the hypothalamus and negative feedback inhibition. Therefore, mutated dogs have different cortisol expressions than other animals. In their urine, several of the cortisol metabolites have been lowered, which was mainly expressed in allo-tetrahydro-cortisol and β-cortol. As a result, the adrenal glands produce less cortisol, leading to lower urinary cortisol metabolite levels [58].

Veterinary visits are an essential part of ensuring a dog’s health and well-being, yet they often present significant stress. The unfamiliar surroundings, the presence of other animals, and handling by strangers can be overwhelming, triggering the activation of the HPA axis. The location of the saliva sample (at the clinic vs. at home) significantly influenced the salivary cortisol levels [25]. Some medical procedures are demanding, physically or physiologically. MRI scans can be insanely stressful for dogs undergoing the procedures, mainly due to loud and unpleasant noises. In dogs without hearing protection, the salivary cortisol levels significantly increased during the MRI scan, even when dogs were put under anaesthesia. In contrast, those with hearing protection had stable cortisol levels. Using hearing protection during MRI scans may help reduce stress and maintain stable physiological responses. Given the high noise levels during MRI procedures, hearing protection is also recommended to prevent potential hearing damage in dogs [59]. Ovariohysterectomy is an invasive procedure, which could potentially lead to increased cortisol levels due to the recovery period after operation, but in some animals, castration was found to lower cortisol levels. In bitches that undergo said procedures, the cortisol/creatinine ratio in their urine reduced significantly since the morning before surgery and stayed at low levels during the study [60]. Aqua-acupuncture, which was expected to alleviate stress, did not significantly affect dogs’ salivary cortisol concentrations during veterinary visits [61]. If the dogs are habituated to semen collection procedures, it does not impact the level of cortisol in their blood. Dogs do not produce cortisol spikes during semen collection as it happens in other species, probably due to the low stress experienced during the procedure. Even female pheromones did not cause cortisol concentrations to increase. During mating naturally, the HPA axis response may differ from the one in clinical settings [62].

While comparing the urinary cortisol metabolite (UCM) concentrations between wolves and dogs, it was expected to find higher UCM in wolves, based on the “selection for tameness” hypothesis in dogs’ domestication. However, dogs had higher UCM concentrations. The effect could be tied to a higher resting metabolic rate in dogs [63].

Although dietary supplements are often a part of behavioural therapies in dogs that are aimed to alleviate stress levels, the food supplementation in tryptophan, beet pulp, salmon oil, soy lecithin, and green tea had no effect on the salivary cortisol concentrations or behaviour on healthy, not prone to anxiousness dogs [6].

#### 4.2.6. Behaviour

Cortisol plays a crucial role in shaping a dog’s behaviour by regulating how they respond to the environment, since it prepares the body to cope with threats. While it is essential for survival, it can significantly influence a dog’s mood, behaviour, and overall well-being. There is a significant positive correlation between HCC in samples collected at 9:00 h and the dog’s latency to resume feeding after the playback of the sounds of a thunderstorm and the reactivity to the thunderstorm. Dogs with higher hair cortisol levels exhibited stronger reactions to those sounds. Those with elevated cortisol levels were likelier to display behaviours like hiding, running away, panting, and lowering their body posture. Measuring cortisol in hair can be a reliable biomarker for assessing a dog’s overall stress levels and temperament [10]. In the dogs that showed behavioural responses to the storm recording, the salivary cortisol levels significantly increased 20 min after the tape was played. Stress response to the storm sounds increased the saliva cortisol levels two times more than the baseline—from ~1.0 ng/mL to ~2.0 ng/mL [3]. Surprisingly, lower hair cortisol concentrations levels were shown in dogs with higher non-social or stranger-directed fear scores. These findings can be explained by suppressing the HPA axis [53]. One of the tests that aims to arouse negative emotions to check the dog’s behaviour is the Dutch Socially Acceptable Behaviour (SAB) test, which is a tool designed to identify aggression and fear in dogs; therefore, it should be physically demanding. The salivary cortisol levels did not significantly change before and after the test, which indicates that it does not raise severe stress levels [7]. In young adult dogs, higher salivary cortisol concentrations 10 min after the behavioural test conducted at home were linked to more desirable characterological traits, as the dogs displayed higher adaptability to the stressors. A long recovery period was linked to negative behaviour, as it could be tied to the difficulty of coping. Those results in puppies could not be linked to their character as young adults, probably due to the impact of character development and socialisation with the environment [64]. In Cavalier Kings Charles Spaniels’ puppies, a rise in salivary cortisol was tied to more avoidance-related behaviour during the behavioural test [45].

#### 4.2.7. Keeping Conditions

The conditions in which dogs are kept play a critical role in their well-being, directly influencing their responses to stress and influencing the dog’s ability to cope. In South Korea, dogs farmed for meat are primarily kept in poor environmental conditions, since their conditions are not regulated by law. Their cortisol levels (40.81 ± 4.58 ng/mg) were twice as high as those in dogs kept as companion dogs [8]. More austere keeping conditions are also tied to increased cortisol/creatinine levels in urine [1].

Keeping conditions can have a significant impact on dogs’ salivary cortisol levels. The highest cortisol levels in saliva were found in shelter dogs, while pet companions scored the lowest. Dogs kept in pens had mediocre cortisol levels; in those individuals, castrated males and spayed females had lower cortisol levels than intact ones, probably due to hormonal changes [49]. Factors such as unfamiliar surroundings, limited social interaction, and the duration of stay may contribute to elevated cortisol levels. Shelter dogs exhibited the highest hair cortisol concentrations among the groups, which aligns with the idea that the shelter environment is a significant stressor. Rehomed dogs had significantly lower hair cortisol concentrations than shelter dogs, suggesting they adapted well to their new environment over time. Shelter dogs varied in their duration of stay (ranging from one day to a year). Dogs recently admitted to the shelter exhibited cortisol levels more indicative of recently lived-through stress, while others might have reflected partial adaptation [65]. Shelter dogs’ cortisol/creatinine and serotonin/creatinine urine ratios fluctuate daily. The dogs’ social exposure also influences those markers. Even something insignificant from the human perspective, like putting on a muzzle, could be very stressful in dogs, increasing cortisol levels [35]. On the contrary, in Walker’s et al. [29] paper, dogs kept in the shelter for long and short periods and pet dogs did not have significantly different salivary cortisol concentrations. The lack of differences could be caused either by the small sample size or the dog’s ability to cope with the shelter environment after a few days rather than by the absence of heightened stress in sheltered dogs. Purebred dogs kept in breeding kennels had similar salivary cortisol values to those shown in Table 1—~1.5–2.5 ng/mL [30].

During a stay at animal hotels, dogs experience a peak in salivary cortisol levels on the admission day. With time, the cortisol levels decrease, which suggests the animals’ ability to adapt to the environment. Despite that, animals’ cortisol levels were higher during their 3-day stay at the hotel (median: 2.7 ng/mL, 2.1 ng/mL, and 1.9 ng/mL for each day, respectively) than in the majority of the company dogs, as shown in Table 1. Small dogs had higher salivary cortisol levels, which may suggest their higher susceptibility to environmental stressors [47].

Clinics are incredibly stressful environments for dogs due to the new environment, disorientation after surgical procedures, and pain. Despite disorientation, sedated dogs had lower cortisol concentrations than non-sedated ones. Some behaviours, like lip-licking and panting, correlate positively with increasing salivary cortisol. Head resting was tied to lower cortisol rates, which could signify a relaxed state [66].

Other variables, like hours walked daily, did not significantly influence hair cortisol levels [11].

#### 4.2.8. Working Dogs and Training

Working dogs play a vital role in society. They are trained to perform specialized tasks, such as assisting humans with disabilities or during therapies, conducting search and rescue (SAR) missions, working in law enforcement, or participating in sports. The demands placed on working dogs and the methods of training can influence their behaviour, health, and stress levels.

Actively participating in extensively competitive sports, like flyball, also raised the HCC levels, although the effect was not shown in other competitive disciplines [53]. Dogs that competed in agility courses had slightly heightened salivary cortisol levels after each round but were still in the normal physiological range. During and after competing, dogs showed many stress-related behaviours, which could suggest that competition in that sport could be potentially stressful and arousing [67].

Different training methods could impact the cortisol levels in trained dogs differently. The most stressful situation was ordering the dogs to quit the behaviour, which was a psychological stressor. Physical stimulus, such as using electronic and pinch collars, caused a lower rise in cortisol levels. All of those methods could cause significant stress if misused. Therefore, trainers’ ability to work with those methods should be evaluated before considering using them [68]. After training, dogs trained by aversive methods had higher salivary cortisol levels (mean 2.60 ± 0.50 ng/mL). They presented more avoidant behaviour than the group trained without using those methods (mean salivary cortisol 1.30 ± 0.2 ng/mL). The cortisol levels of the group trained using aversive methods are higher than most of the values in Table 1, indicating that the aversive training may disrupt the dog’s welfare [5]. On the contrary, according to Packer et al. [53], the training methods and tools did not impact prolonged stress markers and therefore had no or low impact on the welfare levels.

SAR dogs, to be certified in Poland, undergo examinations that mimic a post-disaster environment. Participating in those exams causes saliva cortisol concentrations to increase. Rises in cortisol were not dependent on the dog’s efficiency and success during the exam [44]. SAR exams are physically demanding for the dogs; before and after the exams, dogs presented mean salivary cortisol concentrations at 4.2 ng/mL and 4.89 ng/mL, respectively. Those results are higher than most of the results shown in Table 1, which could indicate extensive stress. The emotional state can affect the dogs’ performance during the SAR exams; the higher the cortisol concentrations were, the less successful the pair was during the exams [69].

Dogs participating in animal-assisted activities did not show higher salivary cortisol levels, which suggests that AAA sessions were not stressful for the animals [70]. During animal-assisted intervention, dogs did not face significantly stressful situations; their salivary cortisol levels did not increase after controlled therapy sessions, nor did they in comparison to the samples taken at home. Behaviour considered a sign of stress also did not increase during therapy sessions, indicating that the dogs were not excessively stressed during the procedure. The stress that the dogs experienced during those sessions may be reduced over time due to positive conditioning and habituation [71]. Similar findings were published by McCullough et al. [46], who also found that higher cortisol levels were correlated with showing more stress-related behaviours during sessions, which suggests that cortisol might be a good indicator of stress in therapy dogs. During the animal-assisted therapy courses, dogs’ salivary cortisol concentrations slowly lowered with each day, which might have been tied to the adjustment to the environment [42]. In another study, Haubenhofer et al. [72] found that dogs’ salivary cortisol concentrations increased on therapy days and slightly after the sessions. Prolonged sessions resulted in a cortisol peak after about 3 h.

Service dogs assisting veterans with PTSD have similar chronic stress levels as companion dogs, suggesting that their training and management after being admitted to a new handler may effectively mitigate the potential stressors of their role. The average cortisol levels in the hair of service dogs (9.69 ± 2.77 pg/mg) did not significantly differ from those of companion dogs (8.65 ± 3.09 pg/mg) [11]. According to Gerwisch et al. [43], signalling and companion dogs had similar salivary cortisol levels, whereas PTSD assistance dogs had a decreased level. It is hypothesised that the decrease in cortisol levels is caused by the dogs’ closer relationship with their owners due to the specifications of their work. While other dogs, like one of the control groups, companions, may also have a strong attachment to their humans, it might be tied to more family members. Family dogs also are not as involved in the daily schedules of their owners.

#### 4.2.9. Social Factors

Dogs are social animals with a high need for intra-species contact. Their living conditions have a high impact on the stress they experience. Social contact can potentially decrease experienced stress, leading to lower cortisol levels. Keeping dogs in pairs or group housing reduced the long-term cortisol levels and slightly improved their behaviour, which resulted in less barking and pacing. The average baseline hair cortisol for all dogs was 3144.6 ± 605.9 pg/mg, and in post-intervention pair-housed dogs, the hair cortisol dropped to 693 ± 298 pg/mg. Those levels are exceptionally high compared to other studies from Table 2 [15].

Many factors can influence hair cortisol concentrations. In border collies, higher HCC levels were found in dogs that lived in households with three or more dogs. This effect can be explained by the dogs’ higher arousal (powered by playing more often) or more competitiveness over resources [53]. However, according to van Houtert et al. [11], the presence of other pets/dogs did not significantly influence the hair cortisol levels. Despite that, sharing the same environment may not cause similarities in dogs, their owners, and cats living in the same household, even though levels between dogs and their owners can be codependent based on their close emotional bond due to social synchronisation [9]. Higher cortisol levels were tied to being left alone in single-dog households for a longer period, but that effect did not occur if the household had more than one dog [14].

Dogs and their owners’ salivary cortisol levels were correlated both before and after the SAR exam, which could indicate the dogs’ ability to pick up their owner’s stress. Bitches and their female owners’ cortisol concentrations interactions were more pronounced, which could point to different emotional reactivity and stress sensitivity in female pairs [68]. Training and social sessions with humans can improve the welfare of canids. Dogs had higher salivary cortisol levels than socialised wolves before and after training sessions. The trainer and their behaviour significantly affected the canid’s relaxation, which was probably mostly tied to the tone of voice, body language, and tone of the interaction [73]. Owners who scored higher in neuroticism levels had dogs with lower iCV, indicating that an owner’s emotional stability can impact their dog’s stress responses. A secure owner–dog bond in promoting adaptive stress responses in dogs, stress responses in human–dog pairs are interconnected, with both human and canine personalities, attachment styles, and gender combinations playing roles in their mutual stress regulation. The owner’s personality and attachment style play a significant role in shaping a dog’s cortisol responses [28].

The salivary cortisol levels significantly increased during the separation period (SP), confirming that separation from owners is a source of acute stress in dogs. The cortisol levels were highest during the initial stages of separation (SP1), with a gradual decline over time, likely due to habituation or stress adaptation. Both groups with owner-related stimuli demonstrated significantly reduced stress levels compared to the control group, emphasising the calming effects of owner-associated olfactory and auditory cues [4]. After interacting with their owners for over 30 min, the dogs’ saliva concentration dropped significantly [31]. Despite the popular belief when it comes to noise phobias in dogs, the owner’s behaviour during playing storm playback did not impact the physiological response to the distressing sounds [74].

Despite the individual differences when it comes to social approaches to humans, scheduled sessions of interspecies interactions may be beneficial to dogs’ adjustment and lower the level of cortisol in their saliva. Dogs that did not have human contact scheduled had high cortisol levels on the third day of their stay in the shelters. Even the dogs with human contact sessions had approximately higher cortisol levels than most of the results of companion dogs presented in Table 1—approximately 2.52–3.14 ng/mL [41]. The stroking or isolation test did not affect the dog’s HPA axis activity. It should be taken into account that those dogs were exceptionally socialised. Therefore, those situations might have less impact than on other animals [75]. Dogs secluded into being insecurely and securely attached to their owners responded differently to the strange situation procedure. Insecure dogs had significantly higher salivary cortisol concentrations than secure dogs after strange situation procedures. Even before the test, insecure dogs had slightly higher cortisol values, which could indicate that those dogs had elevated stress response levels. Those findings did not occur in hair cortisol concentrations, which suggests that attachment style was not tied to chronic stress [76]. Animals admitted to shelters or caught as strays had higher hair cortisol concentrations than most of the pet dogs. Petting sessions, even as short as 15 min, have shown potential in reducing plasma cortisol levels. The effect was especially pronounced in stray dogs—owner-relinquished dogs showed less cortisol reduction, possibly due to the added stress of abrupt changes in their environment and attachment disruption [77].

The litter size highly influences hair cortisol levels in birthing dams, but in bitches that had positive human interaction enrichment provided, the increase was lower [78]. It highlights the positive impact of pleasant interactions with people in a dog’s life, even in that vulnerable period.

## 5. Conclusions

There is a noticeable shift in cortisol research towards non-invasive sampling methods, which aligns with research ethics. These approaches are reliable for assessing both acute and chronic stress responses while maintaining reliable results and easier storage in some samples. Unfortunately, current research is often limited by small sample sizes and variability in methodologies, which remain challenging. Cortisol levels may be influenced by genetic, environmental, health, and social factors. The findings highlight the need for developing standardised sampling techniques, more extensive studies, sensitive methods of assessment, and cross-matrix comparisons to improve the reliability of findings, as well as interdisciplinary approaches, to further understand cortisol’s role in canine welfare. Cortisol measurements, when combined with behavioural observations, may offer a powerful tool to assess stress and overall welfare levels. This approach accounts for individual variability and captures physiological and psychological stress dimensions. Pilot studies are recommended to optimise the protocols for joint behavioural and physiological monitoring. In that case, the calming signals in dogs and a standardised ethogram might be advantageous. Adequately trained staff could analyse body language using a predefined scoring system. This approach could improve the understanding of dogs’ stress dynamics. Future research should focus on refining non-invasive sampling techniques and exploring cortisol variability as an indicator of adaptive stress responses.

In future research, standardised sampling times, conditions, and body parts of the sample taken (in hair samples) should be implemented. Strict protocols must be developed for sample handling, storage, and transportation. The personnel responsible for sample taking and analysis should be thoroughly trained in the following protocols. All research should include detailed descriptions of sample collection, handling, storage, and assay procedures to allow other researchers to replicate the study and compare results accurately. Researchers should also provide results using standardised, consistent units to facilitate study comparison, use thoroughly validated assay methods, and provide quantitative evaluations.

## Figures and Tables

**Table 1:** Values of cortisol in the saliva of companion and shelter dogs.

Saliva		
Companion Dogs	Shelter Dogs	References
1.72 ± 1.88 ng/mL	-	[3]
5.90 ± 1.30 ng/mL	-	[4]
Group Aversively Trained: 1.50 ± 0.2 vs. Group Mixed: 1.4 ± 0.2 vs. Group trained using Reward: 1.30 ± 0.2 ng/mL	-	[5]
0.83 ± 0.22 ng/mL	-	[6]
0.98 ± 0.14 ng/mL	-	[7]

**Table 2:** Values of cortisol in the hair of companion and shelter dogs.

Hair		
Companion Dogs	Shelter Dogs	References
18.83 ± 1.35 ng/mg	-	[8]
0.26 ± 0.12 ng/mL	-	[9]
180.8 + 21.49 pM/g–264.89 + 26.40 pM/g	-	[10]
8.65 ± 3.09 pg/mg	-	[11]
9.5 ± 11.1 pg/mg	-	[12]
5.65 ± 1.28 pg/mg	-	[13]
17.48 ± 8.95 pg/mg	-	[14]
-	1195 ± 555 pg/mg	[15]

**Table 3:** Values of the cortisol–creatinine ratio in urine of companion and shelter dogs.

Urine		
Companion Dogs	Shelter Dogs	References
4.8 ± 0.5—cortisol to creatinine ratio	-	[1]

**Table 4:** Values of cortisol in claws from dead puppies.

**Claws**	
**Puppies (Dead)**	**References**
62.6 ± 58.8 pg/mg	[16]

**Table 5:** Values of cortisol in foetal fluids of dogs.

Fetal Fluids		
Allantoic Fluids	Amniotic Fluids	References
-	5.9 ± 3.0 ng/mL in puppies delivered by ELCS; 10.7 ± 4.2 ng/mL in puppies delivered by EMCS	[17]
-	4.8 ± 4.0 ng/mL	[18]

## Data Availability

The raw data supporting the conclusions of this article will be made available by the authors on request.
